# 

**Published:** 1914-05-09

**Authors:** 


### CONTENTS.

PAGE

**The Strength of Metropolitan Voluntary Hospitals** ... ... ... ... ... ... ... 141

**Treatment by Tuberculin** ... ... ... ... ... 142

**Hospital and Institutional News** ... ... ... ... 143

The Superintendentship at St. George's—The New Budget and the Hospitals—Premier's Cousin Resigns a Medical Post—The Control of the Workhouse Master—The Mental Hospital Chapel—The Relation of Environment to Worship—A New Colony for the Feeble-minded—A Royal Birthday Cake for Crippled Children—Prison Refinements, Old and New—Salvarsan and Lambeth Infirmary—New Medical Superintendent of Downs Sanatorium—A Hospital's Equipment—The Legal Relation of Guardian and Medical Officer—Is Ward Ventilation a Medical Matter?—A Measure of Sanatorium Benefit—The New Principal of Livingstone College—London Midwives' Inspector Honoured—Paying Patients at Tuberculosis Dispensaries—Throat Hospitals Amalgamation—Coal Saving at a Mental Hospital—The Relation of Ventilation to Oratory—The Medical Examination of Workhouse Admissions—Press Reporters in a Hospital Kitchen—The Blind as Actors, Authors, and Musicians—Arthritis Research at Buxton—Chairman's Gift of a New Hospital—A Sea-voyage as a "Cure" for Cancer—The Marriage of Faith and Science—This Week's Drug Market.

PAGE

[*See over.*]

### CONTENTS—(*continued*).

PAGE

**The Treatment of Cancer of the Cervix Uteri** ... ... ... ... ... ... ... 149
Wertheim's Operation, the Bumm and Werder Methods.

**Research and Remedies** ... ... ... ... ... 150

The Passing of Parasyphilis—The Latest Treatment for Eclampsia—Neo-Salvarsan for Scarlet Fever.

**Reports on Hospitals of the United Kingdom**— 151
By SIR HENRY BURDETT, K.C.B., K.C.V.O
West Kent General Hospital, Maidstone.

**Public Pharmacists' Bohemian Concert** ... 153

**Gifts and Bequests** ... ... ... ... ... ... 153

**Round the World** ... ... ... ... ... ... 154

Fellow-Workers and their Doings.

**National Insurance Act Developments** ... ... 155
The National Insurance Act (Part II.) Amendment Bill.

**King Edward's Hospital Fund for London** ... 157
Annual Meeting of Governors and General Council.

**Personalia** ... ... ... ... ... ... ... 159

PAGE

**The Editor's Letter-Box** ... ... ... ... ... 160
Non-Panel Men and the "Medical Directory"—Imperial Health Conference—A Disclaimer.

**Death at South Shields Union Infirmary** ... 160

**The Practical Equipment of a Hospital: Suggestions for an Ideal Locker** ... ... ... ... 161
Our First Competition—The Six Selected Designs—The Voting Paper and the Prizes—How to Vote.

**The Patients' Column** ... ... ... ... ... 165

In an Urban District Infectious Hospital.

**Book Reviews in Brief** ... ... ... ... 165

**The National Medical Union (Official)** ... ... 166
Meeting of Executive Committee.
Sanatorium Benefit Statistics.

**The Institutional Worker** ... ... (Suppl.) 1
The Water Supply from Wells: Selected Answers.
A Sad Case.
Questions for May.
Questions Invited.
Employment and Training.
Practical Matters.
Miscellaneous.

